# Functional connectivity density alterations in children with strabismus and amblyopia based on resting-state functional magnetic resonance imaging (fMRI)

**DOI:** 10.1186/s12886-021-02228-3

**Published:** 2022-02-02

**Authors:** Yi-Dan Shi, Qian-Min Ge, Qi Lin, Rong-Bin Liang, Qiu-Yu Li, Wen-Qing Shi, Biao Li, Yi Shao

**Affiliations:** Department of Ophthalmology, The First Affiliated Hospital of Nanchang University, Jiangxi Province Ocular Disease Clinical Research Center, No 17, YongWaiZheng Street, DongHu District, Nanchang, 330006 Jiangxi People’s Republic of China

**Keywords:** Strabismus, Amblyopia, Magnetic resonance imaging

## Abstract

**Purpose:**

To explore functional connectivity density (FCD) values of brain areas in children with strabismus and amblyopia (SA) based on blood oxygen level-dependent (BOLD) signals.

**Methods:**

This study recruited 26 children (14 male, 12 females) with SA and 26 healthy children (14 male, 12 female) as healthy controls (HCs). Both groups matched in age, gender, educational level and socioeconomic background. While resting, all participants underwent fMRI scanning and global FCD (gFCD) and local FCD (lFCD) values were calculated. Receiver operating characteristic (ROC) curves were created to investigate whether there was a significant difference between children with SA and healthy controls.

**Results:**

When compared with healthy controls, children with SA had significantly lower gFCD values in the right cerebellum, left putamen, and right superior frontal gyrus; however, the same metrics showed opposite changes in the right angular gyrus, left middle cingulate gyrus, left angular gyrus, right superior parietal gyrus, and right middle frontal gyrus. In children with SA, lFCD values were found to be remarkably decreased in regions of the middle right temporal pole, right cerebellum, left putamen, left hippocampus, right hippocampus, left thalamus, left cerebellum; values were increased in the right superior parietal gyrus as compared with healthy controls.

**Conclusion:**

We noted abnormal neural connectivity in some brain areas of children with SA; detailing such connectivity aberrations is useful in exploring the pathophysiology of SA and providing useful information for future clinical management.

## Introduction

Both strabismus and amblyopia (SA) are widespread ophthalmology conditions which often manifest in infancy. Extraocular muscle (EOM) dysfunction is believed to be among the primary etiologies of this condition, as is dysplasia, malnutrition and abnormal anatomy [1]. Such pathology is associates with maldevelopment of visual pathways responsible for mediating eye movement. If no timely and effective treatment is provided, symptoms persist into adulthood [2, 3]. Patients suffering from strabismus cannot grasp synchronized binocular vision as the visual axis of each eye remains in misalignment with that of the contralateral eye. Such symptoms, in turning, lead to damage of visual acuity. Common complications included amblyopia and stereoblindness. Strabismus frequently causes amblyopia, while amblyopia results in perceptual strabismus without effective intervention. Accidents in SA are thought to have increased with develops society. The incidences of strabismus and amblyopia in eastern, and the whole of China, are 5.65 and 1%–3%, respectively [4]. Strabismus not only affects vision in childhood, but also threatens psychological health, social functioning and early character formation [5–7]..

Magnetic resonance imaging (MRI) is a noninvasive technique drawing the anatomical structure and functional alterations of the brain [8]. In particular, blood oxygen level-dependent (BOLD) fMRI as an MRI-based technique allows for measuring of brain function based on variations in deoxyhemoglobin levels during spontaneous brain activity in specific brain regions, which include resting-state functional MRI (rs-fMRI) and task-related fMRI (tr-fMRI) [9]. Compared to tr-fMRI, rs-fMRI does not require the subject to perform a specific performance during the MRI scan, offering a higher degree of acceptance and manoeuvrability. However, rs-fMRI cannot visualise the functional connectivity (FC) between different brain regions, therefore functional connectivity density (FCD), which can compensate for this drawback, was chosen for this study [10].

FCD mapping, which includes both global (gFCD), short-range (or local; lFCD) and long-range FCD mapping, represents functional data of the entire brain using voxel-based morphometry. The lFCD primarily reflects the functional connectivity of clusters over anatomically short distances, and more broadly, the gFCD encompasses both the lFCD and the long FCD, a highly active FCD may be suggestive of this brain region acting as a hyper sensitive functional center [11]. Neuronal apoptosis, development, remodeling, and degeneration all result in changes in FCD, which has previously been used in research studying Parkinson’s disease [12], Alzheimer’s disease [13], and depression [14].

Prior studies reported that patients suffering amblyopia also suffered damage to multiple functional brain areas, as shown by white matter and gray matter morphology aberrations [15]. Lesions were not only observed in the visual cortex as well as the dorsal and ventral visual pathways, but also in higher-level brain areas vital to visual attention and cognition; alterations in functional connectivity among several brain areas instead of isolated, noticeably localized changes were thus found to be hallmark [15]. Here, we calculated gFCD and lFCD values based on BOLD signals, exploring changes in spontaneous brain functional activity of children suffering SA.

## Subjects and methods

### Subjects

The SA group consisted of 26 children who had sought medical care at the Eye Clinic of First Affiliated Hospital of Nanchang University (Nanchang, China) and included 14 males and 12 females. Inclusion criteria were as follows: 1) children < 18 years old; 2) children diagnosed with strabismus; 3) children possessing a difference of > 2 lines (≥0.20 logMAR units) between the best-corrected visual acuity of the amblyopic eye and uncorrected acuity in the unaffected eye as measured with subject view fixed straight ahead. Exclusion criteria were as follows: 1) a history of ocular surgery; 2) a history of other ophthalmology conditions; 3) the presence of conditions precluding rs-fMRI imaging, including mental conditions; 4) drug or alcohol addiction. Twenty-six healthy children who matched study subjects in age, sex, educational level and socioeconomic backgrounds were recruited into a healthy controls (HCs) group. Criteria for HC group recruitment were a lack of MRI findings attributable to cerebral parenchymal pathology, no history of ophthalmologic illness and no history of mental conditions (e.g. Depressive psychosis, epilepsy). The visual acuity of all subjects was evaluated by Snellen chart. Subjects were examined with the horizontal distance between them and the vision chart maintained at 5 m; their line of sight remained fixed parallel to the 1.0 mark on the chart. This study was approved by the Medical Ethics Committee of the First Affiliated Hospital of Nanchang University and was conducted under the Helsinki Declaration.

### MRI scanning

A Trio 3.0 T MRI machine (Siemens, Germany) was used in this study and 8-channel head coils were used to obtain MRI images of the head; rs-fMRI data was acquired by echo planar imaging (EPI) sequence with the following parameters: TR/TE = 2000 ms/30 ms; flip angle (FA) = 90°; thickness = 4 mm; gap = 1 mm; layers = 33; matrix = 64 × 64; field of view (FOV) = 240 mm × 240 mm; containing 240 time points. High resolution structural phase data was acquired using the T1WI 3D MP-RAGE sequence with the following parameters: TR/TE =1900 ms/2.26 ms; FA = 9°; thickness = 1 mm; gap = 0 mm; layers = 156; matrix = 256 × 256, FOV = 240 × 240 mm; scanning time = 3 min. During MRI scanning, subjects were mandated to lie still and avoid movement as best as possible while keeping eyes closed, staying conscious and remaining relaxed. Subjects were instructed not to consider anything in particular during imaging.

### Calculations

MRIcro Software (www.MRIcro.com) was used to ensure high-quality data organization. The first 10 images were discarded to allow for equilibration and subject adaptation. Brain imaging data processing and analysis toolbox (DPABI 2.1, MathWorks ABI) based on MATLAB 2010a (MathWorks, http://rfmri.org/DP, USA) was adopted to precondition remaining data. All subjects remained still until scanning was completed. With reference to the Friston 24-parameter model, multiple linear regression analysis (6 head motion parameters, 6 head motion parameters from the previous time point, and 12 corresponding square items) was used to correct head movement and avoid issues such as inaccurate positioning due to head movement. Extraneous variables, which include respiration, white matter and cerebrospinal fluid signals, were subsequently eliminated via linear regression modeling. The Montreal Neurological Institute standard template was invoked as a reference image for spatial normalization when correcting for head motion. All voxel time series were band-pass filtered (0.01 ~ 0.08 Hz) and data attenuated for both maximal accuracy and minimization of interference factors (e.g. heartbeat, respiratory noise).

### Functional connectivity mapping

The FCD mappings was selected for the grey matter (GM) assessment across all subjects both gFCD and lFCD maps were constructed through the method described by Tomasi and Volkow [10] to calculate values using an internal script. Based on the Pearson correlation coefficient, the number of functional connections of a prescribed voxel was regarded as the degree of node in the binary graph. Voxels with correlation threshold (r) > 0.25 were considered, and those with anatomical distances ≤14 and > 14 were defined as lFCD and gFCD, respectively. The BrainWaver toolbox (http://cran.rproject.org/src/conTRIB/Archive/Brainwaver) was used to calculate gFCD and lFCD values. Z-scores were carried out on the gFCD and lFCD values to conform more closely to the normal distribution. Statistical parameters were finally utilized for mapping in SPM8 (https://www.fil.ion.ucl.ac.uk/spm/software/spm8/) and a 6 × 6 × 6 mm^3^ Gaussian kernel was used to spatially smooth the normalized gFCD and lFCD map.

### Correlation analyses

This study used SPSS 20.0 software (SPSS, USA) to evaluate the relationship between abnormal brain functional areas in children suffering SA and anxiety or depression. This study used the Hospital Anxiety and Depression Scale (HADS) to evaluate the levels of anxiety and depression experienced by our subjects [16]. Participants were inquired as to their psychological status over the prior month. Maximum score for anxiety and/or depression was 21 and it is considered that the patient is in a state of anxiety or depression when the score ≥ 8.

### Statistical analyses

Differences among SA and HC groups were analyzed using SPSS 20.0 software (SPSS, USA) and the independent t-test (*P* < 0.05). Here, we detected discrepancies of gFCD and lFCD averages among SA and HC group subjects using SPM8 software (https://www.fil.ion.ucl.ac.uk/spm/software/spm8/) by applying a general linear model and the double sample t-test. Age and gender were used as covariates in the evaluation of cerebral blood flow graph differences among SA patients and HC subjects; Gaussian random field theory (GRF) correction was used as the significance criterion for data correction (voxel level *P* < 0.01, cluster level *P* < 0.05).

Furthermore, based on the results of previous studies. It is reasonable to speculate that variations in patients’ gFCD and lFCD values may have some diagnostic aid because of their statistical and clinical significance and may be used to differentiate patients from HCs. Therefore, the area under the curve (AUC) in the ROC curve was chosen for this analysis because both sensitivity and specificity could be considered. Values of 0.5–0.7 AUC imply low accuracy; 0.7–0.9 means average accuracy; greater than 0.9 signifies high accuracy.

## Results

### Subject data

No significant differences among SA and HC subjects were noted in age (*P* = 0.884); best-corrected left eye (*P* = 0.012) and right eye (*P* = 0.007) acuity significantly differed (Table 1).

**Table 1:** Participant characteristics

Condition	SA	HCs	t	*P*-value*
Male/female	14/12	14/12	N/A	> 0.999
Age (years)	8.21 ± 2.12	8.38 ± 1.86	0.259	0.884
Weight (kg)	20.58 ± 2.98	21.03 ± 3.74	0.462	0.817
Handedness	26R	26R	N/A	N/A
Duration of SA (years)	7.01 ± 2.32	N/A	N/A	N/A
BCVA-left eye	0.20 ± 0.05	1.00 ± 0.15	−3.158	0.012
BCVA-right eye	0.50 ± 0.15	1.10 ± 0.10	−3.653	0.007
IOP-L	14.65 ± 4.16	15.12 ± 4.03	0.696	0.853
IOP-R	13.46 ± 3.27	14.53 ± 4.11	0.721	0.834

*Notes***:** Independent t-tests comparing the two groups (*p* < 0.05 represented statistically significant differences). Data shown as mean standard deviation or n

*Abbreviations***:** *BCVA* Best-corrected visual acuity, *HCs* Healthy controls, *IOP* Intraocular pressure, *L* Left, *N/A* Not applicable, *R* Right, *SA* Strabismus and amblyopia

### Functional connectivity density analysis

**Fig. 1:**
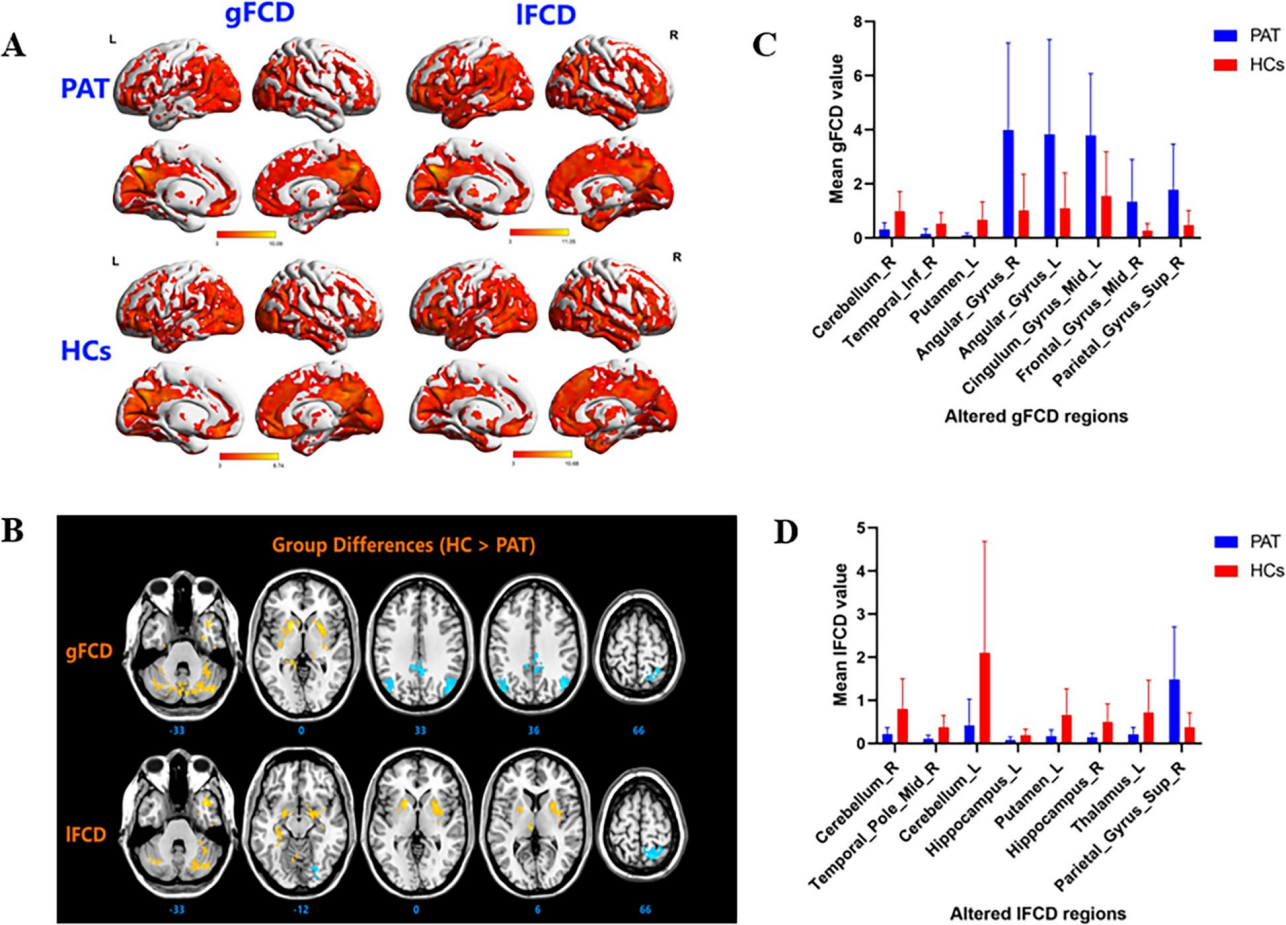
**A** Binarized gFCD (left) and lFCD (right) values in different brain regions. Red and blue bars indicate the HC and SA groups, respectively. **B** The red areas depict higher gFCD and lFCD values. Significant gFCD differences were observed in the right cerebellum, left putamen and right superior frontal gyrus, right angular gyrus, left middle cingulate gyrus, left angular gyrus, right superior parietal gyrus and right middle frontal gyrus. The color bars show the means of altered lFCD between the SA and HC groups. Significant lFCD differences were observed in the middle right temporal pole, right cerebellum, left putamen, bilateral hippocampus, left thalamus and left cerebellum, right superior parietal gyrus. The color bars show the means of altered lFCD between the SA group and HCs. **C** Binarized gFCD and lFCD differences between the HC and SA groups. Yellow areas show lower values. Abbreviations: gFCD, global functional connectivity density; lFCD, local functional connectivity density; PAT, patient; HCs, healthy controls; L, left; R, right

As compared to HC subjects, gFCD values of SA patients were markedly decreased in the right cerebellum, right inferior temporal gyrus and left putamen; meanwhile, they were increased in the right angular gyrus, left angular gyrus, left middle cingulate gyrus, right middle frontal gyrus and right superior parietal gyrus (Table 2; Fig. 1).

**Table 2:** The binarized gFCD differences between the SA and HC groups

Brain areas	MNI coordinates						
	X	Y	Z	BA	Peak voxels	Tvalue	ROI
HC > PAT							
Cerebellum_R	45	−75	−33		1350	4.25	Cluster 1
Temporal_Inf_R	48	−3	−39	20	99	3.87	Cluster 2
Putamen-L	−18	9	0		177	4.17	Cluster 3
HC < PAT							
Angular_Gyrus_R	45	−66	36	39	128	−4.02	Cluster 4
Angular_Gyrus_L	−42	−66	30	39	116	−3.45	Cluster 5
Cingulum_Mid_L	−9	−45	33	31	105	−3.77	Cluster 6
Frontal_Gyrus_Mid_R	42	27	39	8	41	−3.21	Cluster 7
Parietal_Gyrus_Sup_R	30	−54	66	7	47	−3.43	Cluster 8

*Notes*: Between-group differences in binarized gFCD at a threshold of *r* = 0.3. Voxel-wise *P* < 0.01 and cluster-level *P* < 0.05 were used to identify significant group differences, correcting for multiple comparisons by AlphaSim.

*Abbreviations*: *BA* Brodmann’s area, *gFCD* Global functional connectivity density, *MNI* Montreal Neurological Institute, *SA* Strabismus and amblyopia, *PAT* Patient, *ROI* Region of interest

The lFCD values of children with SA were remarkably decreased in areas of the right cerebellum, middle right temporal pole, right cerebellum, left hippocampus, left putamen, right hippocampus and left thalamus; lFCD values were increased in the right superior parietal gyrus as compared with HC subjects (Table 3; Fig. 1).

**Table 3:** The binarized lFCD differences between the SA and HC groups

Brain areas	MNI coordinates						
	X	Y	Z	BA	Peak voxels	Tvalue	ROI
HC > PAT							
Cerebellum_R	30	−81	−48		350		Cluster 1
Temporal_Pole_Mid_R	42	12	−33	38	127	4.5	Cluster 2
Cerebellum_L	−45	−63	−27		35	3.08	Cluster 3
Hippocampus_L	−33	−21	−12	37	143	3.43	Cluster 4
Putamen_L	−18	9	0	34	101	3.8	Cluster 5
Hippocampus_R	27	−6	−9	34	249	4	Cluster 6
Thalamus_L	−6	−21	6		31	3.2	Cluster 8
HC < PAT							
Parietal_Sup_R	18	−60	66	7	80	−4.1	Cluster 7

*Notes*: Between-group differences in binarized lFCD at a threshold of *r* = 0.3. Voxel-wise *P* < 0.01 and cluster-level *P* < 0.05 were used to identify significant group differences, correcting for multiple comparisons by AlphaSim.

*Abbreviations*: *BA* Brodmann’s area, *lFCD* Local functional connectivity density, *MNI* Montreal Neurological Institute, *SA* Strabismus and amblyopia, *PAT* Patient

### ROC curve analysis

**Fig. 2:**
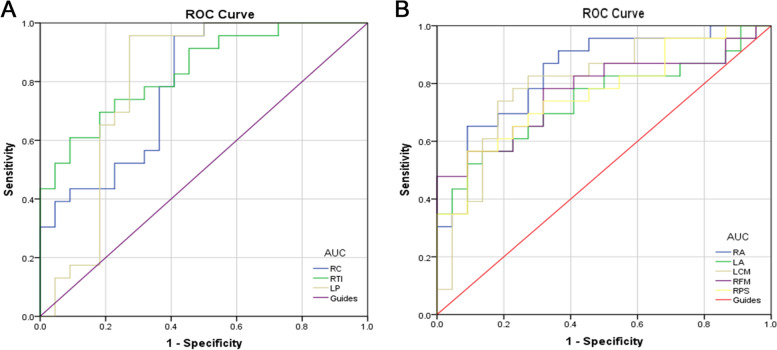
ROC curve analysis of the mean gFCD difference for altered brain regions. **A** The area under the ROC curve were 0.787, (*p* = 0.001; 95% CI: 0.653–0.920) for RC, RTI 0.836, (*p*<0.0001; 95% CI: 0.722–0.950), LP 0.800, (*p* = 0.001; 95% CI: 0.654–0.947). **B** The area under the ROC curve were 0.840, (*p*<0.0001; 95% CI: 0.723–0.956) for RA, LA 0.741, (*p* = 0.006; 95% CI: 0.593–0.889), LCM 0.796, (*p* = 0.001; 95% CI: 0.661–0.932), RFM 0.775, (*p* = 0.002; 95% CI: 0.634–0.915), RPS 0.767, (*p* = 0.002; 95% CI: 0.628–0.905). Abbreviations: AUC, area under the curve; ROC, receiver operating characteristic. RC, right cerebellum; RTI, right temporal inf; LP, left putamen; RA, right angular; LA, left angular; LCM, left cingulum mid; RFM, right frontal mid; RPS, right parietal sup

**Fig. 3:**
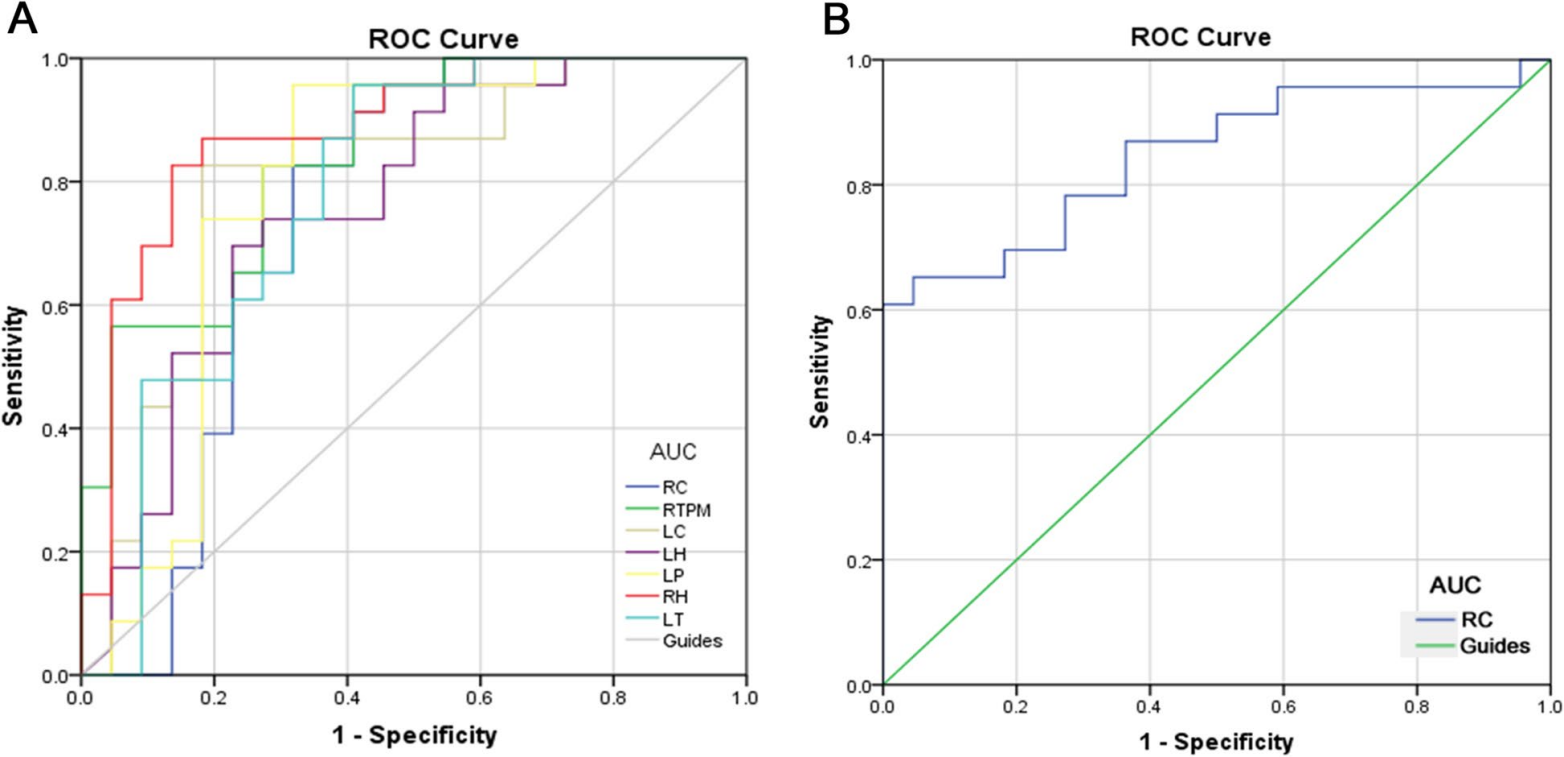
ROC curve analysis of the mean lFCD difference for altered brain regions. **A** The area under the ROC curve were 0.787, (*p* = 0.005; 95% CI: 0.583–0.903) for RC, right cerebellum; RTPM, right temporal pole mid; LC, left cerebellum; LH, left hippocampus; LP, left putamen; RH, right hippocampus; LT, left thalamus; RPS, right parietal sup. Abbreviations: AUC, area under the curve; ROC, receiver operating characteristic. RC, right cerebellum; RTPM, LC 0.800, (*p* = 0.001; 95% CI: 0.663–0.938), LH 0.760, (*p* = 0.003; 95% CI: 0.616–0.904), LP 0.792, (*p* = 0.001; 95% CI: 0.645–0.940), RH 0.881, (*p* = 0.053; 95% CI: 0.778–0.985), LT 0.767, (*p* = 0.073; 95% CI: 0.635–0.922). **B** The area under the ROC curve were 0.846, (*p*<0.0001; 95% CI: 0.730–0.962) for RPS

The AUC values for gFCD were 0.787 for the right cerebellum; o.836 for the right temporal inferior region; 0.800 for the left putamen; 0.840 for the right angular gyrus; 0.741 for the left angular gyrus; 0.796 for the left cingulum mid; 0.775 for the right medial frontal gyrus; and 0.767 for the right superior parietal gyrus (Fig. 2)***.*** The AUC values for lFCD were 0.787 for the right cerebellum; 0.800 for the middle right temporal pole; 0.760 for the left hippocampus; 0.792 for the left putamen; 0.881 for the right hippocampus, 0.881; 0.767 for the left thalamus; and 0.846 for the right superior frontal gyrus (Fig. 3)***.***

### Correlation analyses

Patient anxiety score was found to be 7.23 ± 2.33 while the depression score was found to be 7.63 ± 1.20. Anxiety score was found to negatively correlate in right medial frontal gyrus voxel value (*r* = − 0.845, *P* < 0.001); depression score was likewise finding to negatively correlate with voxel value of that same region (*r* = − 0.842, *P* < 0.001).

## Discussion

Compared with conventional fMRI, rs-fMRI does not require subjects to perform specific actions. This, in turning, allows for more accurate data collection. Compared with the HC subjects, changes in rs-fMRI and FCD results in children suffering SA suggest significant alterations in both patient brain activity and functional connections. Such abnormalities are of major relevance in relation to both the clinical manifestations and pathogenesis of SA and warrant detailed investigation.

**Fig. 4:**
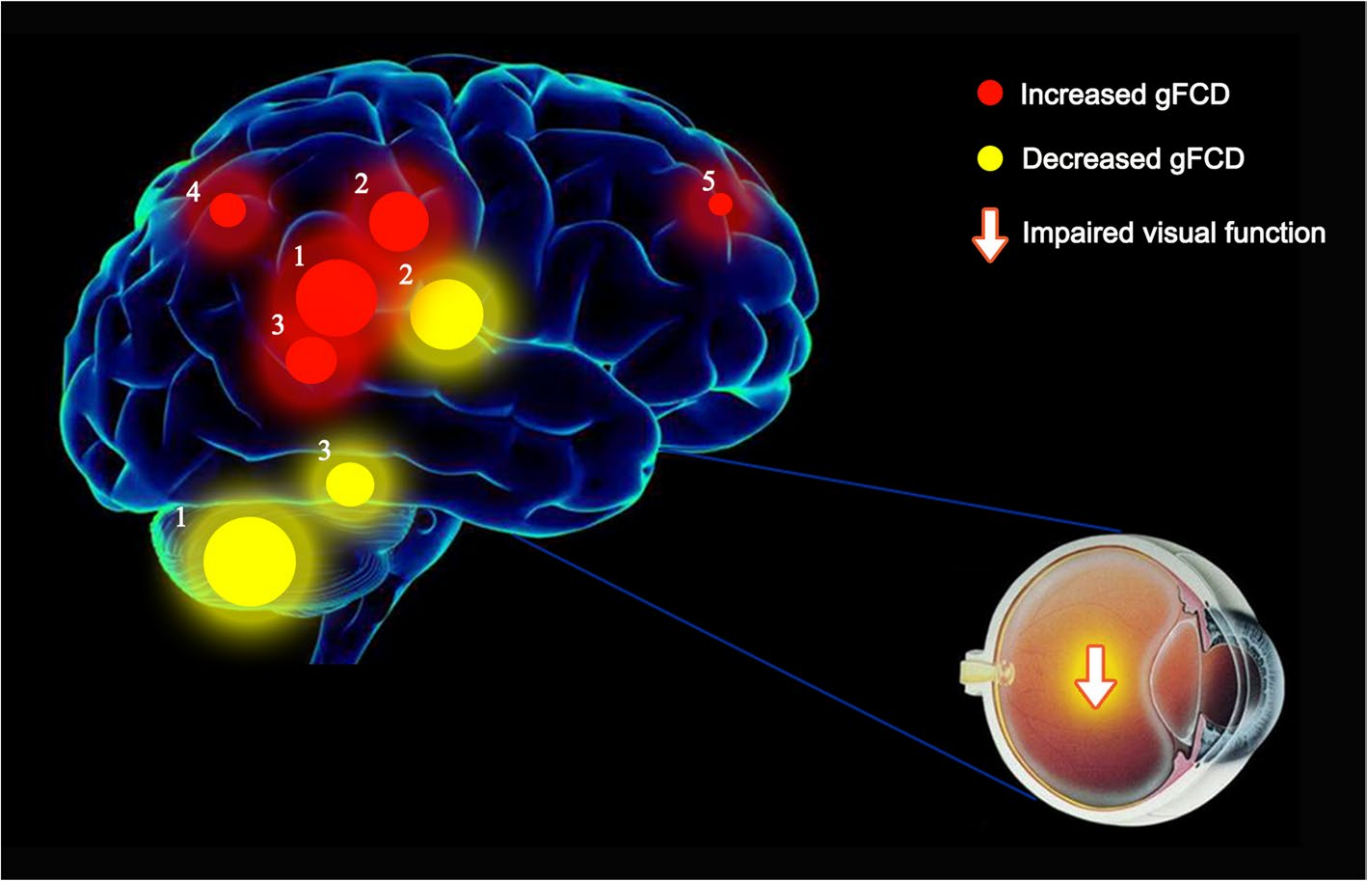
The mean gFCD values of altered brain regions. Compared with the HCs, the gFCD values of the following regions were decreased to various extents: 1- Right cerebellum (t = 4.25), 2- Left putamen (t = 4.17), 3- Right frontal sup (BA 10, t = − 4.27). Compared with the HCs, the gFCD values of the following regions were increased to various extents: 1- Right angular (BA 39, t = − 4.02), 2- Left cingulum mid (BA 31, t = − 3.77), 3- Left angular (BA 39, t = − 3.45), 4- Right parietal sup (BA 7, t = − 3.43), 5- Right frontal mid (BA 8, t = − 3.21). Abbreviations: HCs, healthy controls; BA, Brodmann’s area

**Fig. 5:**
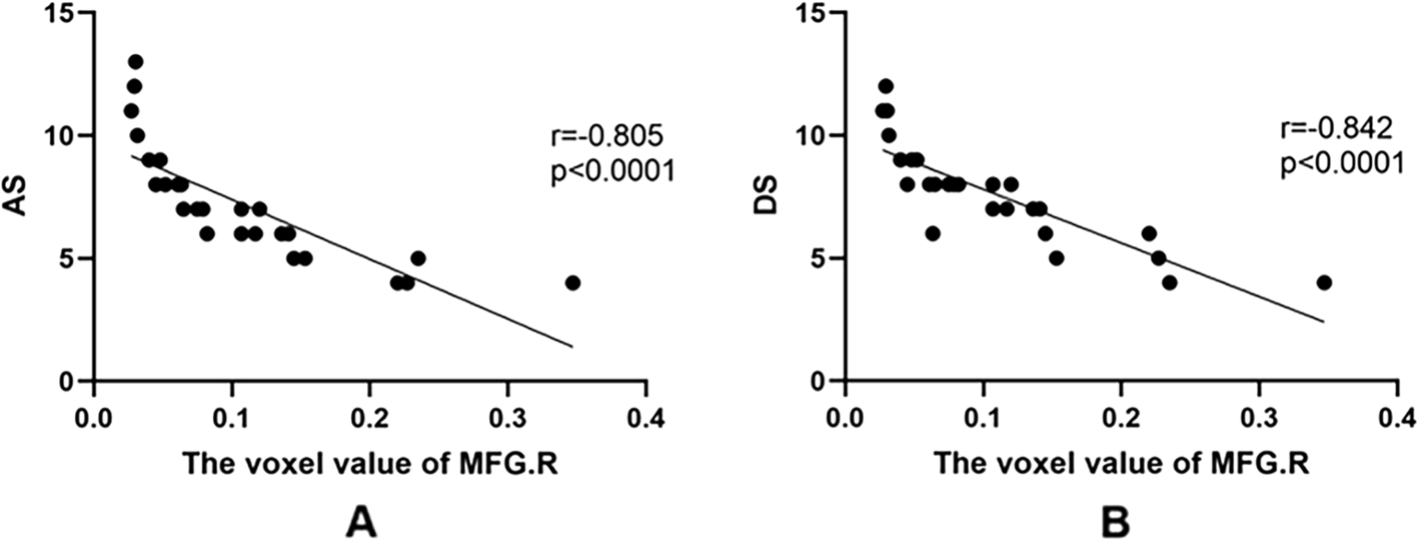
Correlations between the voxel value of right media frontal gyrus and clinical behaviors. **a** The AS showed a negative correlation with the voxel values of right medial frontal gyrus (*r* = − 0.845, *p* < 0.001), and **b** the DS showed a negative correlation with the voxel values of right medial frontal gyrus (*r* = − 0.842, *p* < 0.001). Abbreviations: MFG. R, right media frontal gyrus; AS, anxiety scores; DS, depression scores

The frontal gyrus is widely involved in many physiological functions and its complexity far exceeds that of other central nervous system components. Anatomically, the precentral, superior frontal and inferior frontal sulci divide the frontal lobe into the precentral, superior frontal middle frontal and inferior frontal gyri. These regions all have distinct functions. Disorders of voluntary movement, language, and autonomic functions have been ascribed to frontal gyrus lesions. The frontal gyrus additionally plays a critical role in the maintenance of normal visual function. Previous studies reported SA patients to suffer abnormalities in frontal gyrus function. Patients suffering concomitant exotropia were found to have decreased left frontal gyrus volumes and decreased middle inferior gyrus gray matter density [17]. The frontal eye field, located at the caudal end of the middle frontal gyrus, is vital in the control of eye movement and affects eye movement latency [18]. In addition, earlier studies reported the frontal eye field to be pivotal in saccade formation [19, 20]. Xiao et al. found that children suffering amblyopia have reduced gray matter density in the middle frontal gyrus [17]. Ouyang et al. similarly reported precentral gyrus gray matter volume to be reduced in such patients [21]. Furthermore, prefrontal cortex regional homogeneity and FCD were both found to be reduced in patients suffering with anisometropic amblyopia [22]. In this study, as compared with HC subjects, gFCD values of SA patients were found to be decreased in the right superior frontal gyrus and increased in the right middle frontal gyrus (Fig. 4). Correlation analyses revealed that these phenomena influence patient anxiety and depression (Fig. 5). The alteration of gFCD in the right superior frontal gyrus is consistent with previous studies, suggesting that the patient may have decreased functional connectivity due to abnormalities in oculomotor function. The alterations in right middle frontal gyrus are not supported by previous studies and even demonstrate an inverse trend, while the elevation of its gFCD is negatively correlated with both the anxiety and depressive state of the child with SA. We hypothesize, therefore, that the elevation of gFCD in right middle frontal gyrus may be the result of compensatory activity in the early stages of the disease or may be influenced by the patient’s anxiety and depressive state. The angular gyrus is located in the anterolateral parietal lobe and corresponds to Brodmann area 39. This region of the brain is crucial in sensory, visual, and auditory stimuli processing. Here, we found children suffering SA to have higher gFCD in the left and right angular gyrus as compared to HC subjects, likely due to compensatory increases in attention and spatial comprehension.

The thalamus is primarily responsible for relaying sensory and motor signals to the cerebral cortex [23]. Thalamic nuclei play fundamental roles in the relay of sensory information to the cerebral cortex. Notably, the thalamus is involved in the processing of visual information via the retino-thalamic-cortical pathway [24]. Studies have shown that abnormalities of this pathway affect pathogenesis of a number of neurological conditions, such as autism and temporal lobe epilepsy. Gray matter volume in the right thalamus was previously found to be increased in adults suffering strabismus [25]. In our research, reduced thalamic lFCD values were found in SA children, suggesting possible impairment in visual information and motor integration functions.

The cingulate gyrus, a band of cortex surrounding the corpus callosum, is an integral component of the limbic system and the default mode network [26]. The cingulate gyrus establishes functional connections with the hippocampal cortex, medial prefrontal lobe, and temporal lobe cortex [27]. This brain region is closely involved executive functioning and emotional regulation. Prior studies have also focused on the role of cingulate gyrus in the pathogenesis and clinical manifestations of epilepsy [28, 29]. The anterior cingulate gyrus contains afferent thalamic projections [30]. Thus, this brain region likely plays an important role in eye movement and processing of visual information. In a study of patients suffering strabismus, Ouyang et al. observed that the gray matter volume of the right cingulate gyrus in strabismus patients was significantly reduced. On fMRI, the right cingulate gyrus was found to undergo significant activation in amblyopic patients who underwent perceptual learning therapy as compared to pre-treatment data, underscoring that dysfunction of this area plays an important role in the pathogenesis of amblyopia [31]. Here, gFCD values in the right middle cingulate gyrus of SA children were significantly increased as compared with HC subjects, consistent with findings reported previously. Recovery of cingulate gyrus function thus likely plays a compensatory role in treatment and can be considered as a component of future SA management.

**Fig. 6:**
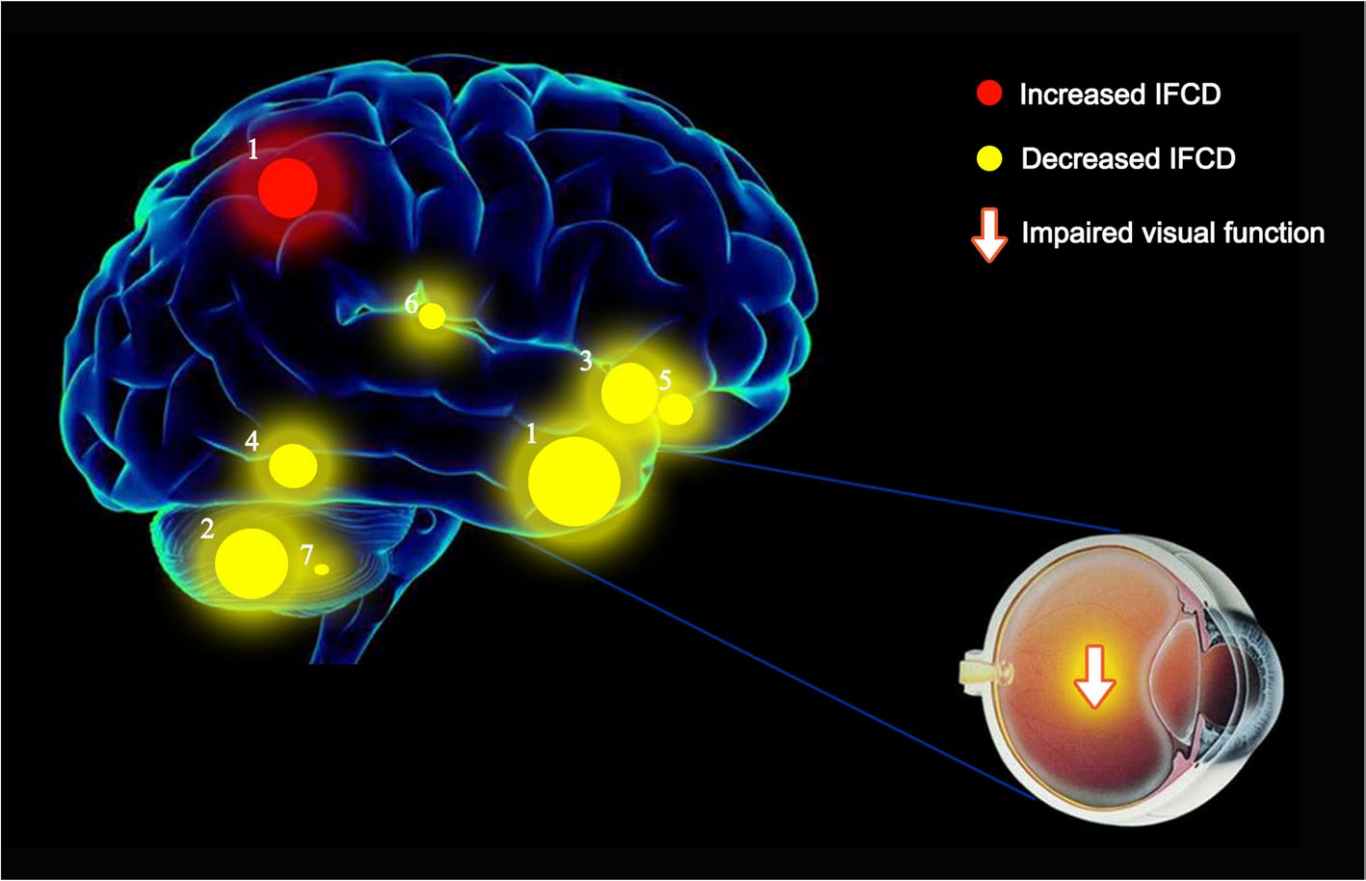
The mean lFCD values of altered brain regions. Compared with the HCs, the lFCD values of the following regions were decreased to various extents: 1- Right temporal pole mid (BA 38, t = 4.50), 2- Right cerebellum (t = 3.90), 3- Left putamen (BA 34, t = 3.80), 4- Left hippocampus (BA 37, t = 3.43), 5- Right hippocampus (BA 34, t = 4.00), 6- Left thalamus (t = 3.20), 7- Left cerebellum (t = 3.08). Compared with the HCs, the lFCD values of the following regions were increased to various extents: 1- Right parietal sup (BA 7, t = − 4.1). Abbreviations: HCs, healthy controls; BA, Brodmann’s area

The parahippocampal gyrus and hippocampus involving in memory, recognition and spatial memory, also interconnecting with the visual system. The parahippocampal region is mainly responsible for memory formation in specific situations; the hippocampus is understood to play a role in long-term memory formation [32]. The anterior hippocampus is furthermore connected to the default mode network, ventral striatum, midbrain and amygdala; these areas play a role in the stress response. The posterior hippocampus is more closely involved in spatial-contextual information processing. Studies have shown that damage to the hippocampus is associated with the development of Parkinson’s disease [33]. When patients suffer from generalized anxiety disorder, their hippocampus volume significantly decreases [34]. McCormick found that the hippocampus and visual areas connect with each other throughout different phases of autobiographical memory formation [35]. In this study, the lFCD values were found to be decreased in the hippocampus bilaterally among SA patients as compared with HC subjects. These findings indicate that SA likely results in impairment of visual-spatial processing due to ocular dysfunction (Fig. 6) (Table 4).

**Table 4:** Brain regions with changed FCD values and its potential impact

Brain region	Brain function	Anticipated results
Cerebellum	Regulating body balance and muscle tone; Coordinating of voluntary movement and language; emotion control; spatial cognition	The damage of spatial cognition
Lenticular nucleus, putamen	Executive ability; language function.	Impairment of font- semantic pathway in visual pathway
Superior frontal gyrus	Cognition; semantic system; episodic memory; indirectly control the sequences of visual-guided saccades and eye–hand coordination.	Reduced spatial cognitive ability; impairment of eye-hand coordination
Angular gyrus	Visual language center	Compensatory enhancement of visual image and auditory image connection
Middle cingulum gyrus	Executive ability; emotional management; visual function	Abnormality of visual function
Superior parietal gyrus	Center of tactile and stereognosis	Stereognosis consumption increased
Middle frontal gyrus	Consist of Frontal eye field	eye movements accuracy consumption increased
Temporal pole mid	Related to high-level social emotional function, implicated in theory of mind	Negative emotion, abnormal social function
Hippocampus	Long-term memory; associated with visual system; spatial information processing capability	The damage of spatial information processing capability
Thalamus	involved in visual sensation; dynamic visual information management	The capability of dynamic visual information processing is impacted

## Conclusion

Through gFCD and lFCD values, we found that children with SA have abnormalities in functional connectivity in certain brain regions, most of which are focused on visual, cognitive and emotion-related pathways. As our sample size was rather small, further research studying a larger patient population is warranted.

## Data Availability

Data used to support the findings of this study are available from the corresponding author upon request.
